# Myxome de la petite valve mitrale: à propos d’un cas

**DOI:** 10.11604/pamj.2017.26.61.11480

**Published:** 2017-02-02

**Authors:** Fouad Nya, Abdessamad Abdou, Mehdi Bamous, Younes Moutakiallah, Noureddine Atmani, Aniss Seghrouchni, Mahdi Aithoussa, Abdellatif Boulahya

**Affiliations:** 1 Mohamed V University, Cardiac Surgery Department, Mohamed V Military Hospital, Rabat, Morocco

**Keywords:** Myxome, petite valve mitrale, tumeurs primitives du cœur, Myxoma, posterolateral leaflet

## Abstract

Les myxomes cardiaques constituent les formes les plus fréquentes des tumeurs primitives du cœur. La localisation la plus fréquente est le septum interatrial et exceptionnellement au niveau des valves cardiaques. L'exérèse chirurgicale est la seule alternative thérapeutique. Nous rapportons l'observation clinique d'un patient âgé de 69 ans, sans antécédents pathologique notables, qui a présenté une dyspnée stade II à III de la NYHA associée à une lipothymie. L'échocardiographie transthoracique a objectivé un rétrécissement aortique calcifié serré avec un gradient moyen VG-aorte à 58 mmHg. Au niveau de la valve mitrale présence d'une masse de 15mm de diamètre, s'insérant sur la petite valve mitrale sessile, sans effet de sténose ni de fuite mitrale évoquant un myxome de localisation atypique ou un fibroélastome. L'examen a été complété par une ETO qui a confirmé le diagnostic de la masse de la petite valve mitrale. Le patient est opéré avec une stérnotomie médiane verticale, et sous circulation extracorporelle conventionnelle. Une atriotomie gauche a permis d'objectiver une masse sessile de 15mm de diamètre sur la face auriculaire de la petite valve mitrale, friable et facilement clivable. Le geste a été complété par une cautérisation de la base d'implantation au bistouri électrique sans geste supplémentaire sur la petite valve mitrale. La pièce opératoire est adressée en anatomo-pathologie qui a confirmé le diagnostic de myxome. Le patient a bénéficié aussi d'un remplacement valvulaire aortique par prothèse mécanique. Les suites opératoires immédiates étaient simples. Le patient a quitté le service à J8 postopératoire. La localisation mitrale du myxome cardiaque est très rare. Le traitement chirurgical reste la seule option thérapeutique avec une résection la plus large possible pour éviter tout risque de récidive.

## Abstract (en)

Cardiac myxomas are the most common type of primary cardiac tumors. They mainly affect the interatrial septum and exceptionally the heart valves. Surgical excision remains the only therapeutic alternative. We here report the case of a 69-year old patient with no significant pathological history suffering from NYHA class II-III dyspnea associated with lipothymia. Transthoracic echocardiography showed a tight calcified aortic narrowing with aortic valve gradient of 58 mmHg. A sessile mass of 15mm diameter inserted into the posterolateral leaflet, without stenosis or mitral regurgitation evoking an atypical localization of myxomaor or fibroelastoma was detected at the level of the mitral valve. The examination was supplemented by ETO which confirmed the diagnosis of a mass involving the posterolateral leaflet. The patient underwent surgery via median sternotomy, under conventional extracorporeal circulation. Left atriotomy allowed to objectify a sessile mass of 15mm of diameter involving the auricular wall of the friable and easily cleavable posterolateral leaflet. Cauterization of the implant base via electric scalpel was then performed without any additional gesture on the posterolateral leaflet. Anatomopathologic analysis of the surgical specimen confirmed the diagnosis of myxoma. The patient also underwent aortic valve replacement with mechanical prosthesis. The postoperative course was uneventful. The patient was discharged on postoperative day 8. Cardiac myxoma involving the mitral leaflet is very rare. Surgical procedure attempting to resect the widest possible surgical margins remains the only therapeutic option to avoid the risk of recurrence.

## Introduction

Les myxomes cardiaques constituent les formes les plus fréquentes des tumeurs primitives du cœur. Leur diagnostic a largement bénéficié de l'apport de l'échocardiographie transthoracique et transoesophagienne. La localisation la plus fréquente est le septum interatrial et exceptionellement au niveau des valves cardiaques. L'exérèse chirurgicale est la seule alternative thérapeutique.

## Patient et observation

Il s'agit d'un patient âgé de 69 ans, sans antécédents pathologique notables, qui a présenté une dyspnée stade II à III de la NYHA d'aggravation progressive associée à une lipothymie. L'examen clinique a trouvé un patient en bon état général, les bruits du cœur sont réguliers, présence d'un souffle systolique au foyer aortique irradiant vers les vaisseaux du cou, l'examen pleuro-pulmonaire et le reste de l'examen somatique sont sans particularités. La radiographie du thorax a montré une silhouette cardiaque de taille normale et une bonne transparence parenchymateuse L'ECG est sans particularités L'échocardiographie transthoracique a objectivé un rétrécissement aortique calcifié serré avec un gradient moyen VG-aorte à 58 mmHg, un VG de taille normale et de fonction systolique conservée. Au niveau de la valve mitrale présence d'une masse de 15mm de diamètre, s'insérant sur la petite valve mitrale sessile, sans effet de sténose ni de fuite mitrale évoquant un myxome de localisation atypique ou un fibroélastome. L'examen a été complété par une ETO qui a confirmé le diagnostic de la masse de la petite valve mitrale. Le patient a bénéficié d'une coronarographie préopératoire qui est normale et le bilan biologique est sans particularités Le patient est opéré avec une stérnotomie médiane verticale, l'installation de la CEC est faite entre une canule artérielle au pied du TABC et deux canules veineuses bi-caves et menée en hypothermie modérée. La protection myocardique est assurée par une cardioplégie cristalloïde froide intermittente antérograde. Une atriotomie gauche derrière le sillon de SONDERGAARD est réalisée permettant d'objectiver une masse sessile de 15mm de diamètre sur la face auriculaire de la petite valve mitrale, friable et facilement clivable. Le geste a été complété par une cautérisation de la base d'implantation au bistouri électrique sans geste supplémentaire sur la petite valve mitrale. La pièce opératoire est adressée en anatomo-pathologie qui a confirmé le diagnostic du myxome de la petite valve mitrale. Le patient a bénéficié aussi d'un remplacement valvulaire aortique par prothèse mécanique. Les suites opératoires immédiates étaient. Le patient a quitté le service à J8 postopératoire constitue le traitement radical et permet une guérison totale et définitive.

## Discussion

La première description autopsique d'un myxome date de 1845 et le premier diagnostic clinique d'un myxome date de 1952 [1]. Il s'agit d'une tumeur rare représentant 0,5 à 1 % des tumeurs des tissus mous. Dans sa localisation cardiaque, le myxome est de loin la plus fréquente des tumeurs cardiopéricardiques primitives bénignes (91 %) [2]. L'origine des myxomes a fait l'objet de multiples hypothèses dont celle du développement à partir de thrombi Préexistants [3]. L'explication communément admise est celle d'une tumeur histologiquement bénigne développée à partir de « reliquats vestigiaux embryonnaires sous-endocardiques pluripotents, séquestrés habituellement dans la région limbique de la fosse ovale » du septum interauriculaire (SIA) [4]. L'oreillette gauche représente 5 à 90 % des localisations des myxomes, l'implantation se fait essentiellement sur le septum interauriculaire, la localisation mitrale est très rare (21 cas décrits dans la littérature jusqu'à 1997) [5]. Le myxome intracardiaque est une pathologie qui se caractérise par un très grand polymorphisme clinique qui peut engendrer un retard diagnostique, bien que les accidents emboliques et les syndromes d'obstruction valvulaire sont les manifestations les plus fréquentes. Il peut être asymptomatique dans 10% des cas [6]. Le diagnostic est aisément évoqué en ETT (une sensibilité de 93,3 % et une spécificité de 96,8 %), l'ETO permet de préciser certaines zones de la tumeur en particulier les zones d'attache. Par ailleurs, le principal diagnostic différentiel est le thrombus intracavitaire, les végétations et les lésions dégénératives du tissu valvulaire. Le traitement chirurgical est la seule alternative opératoire consistant à une résection complète de la tumeur et de sa base d'implantation pour éviter tout risque de récidive, le tissu valvulaire peut être conservé mais en cas de base d'implantation large une excision d'une partie ou de la totalité de la valve peut être réalisée suivie d'une plastie ou d'un remplacement valvulaire. La mortalité précoce est faible inférieure à 5 %. La morbidité tardive est dominée par le risque de récurrence tumorale et la survenue de métastases à distance. La fréquence de ces récidives tumorales est autour de 2 % [7]. Il convient d'insister sur une technique chirurgicale parfaite pour prévenir tout risque de récidive ainsi que sur un contrôle échographique régulier de tout malade opéré pour un myxome cardiaque.

## Conclusion

La localisation mitrale du myxome cardiaque est très rare. La principale complication a redouté est l'embolisation artérielle. Le traitement chirurgical reste la seule option thérapeutique avec une résection la plus large possible pour éviter tout risque de récidive.
